# Assessment of Psychological and Social Impact on Patients Rehabilitated With Conventional and Modern Technology‐Based Maxillofacial Prosthetics

**DOI:** 10.1155/ijod/3504249

**Published:** 2026-10-07

**Authors:** Shahad Fadhil Bunyan, Shahad Mohammed Shakir, Zryan jaza hama salih, Wisam Jasim Mohammed, Quasy Abdulateef Hasan

**Affiliations:** ^1^ Department of Forensic Evidence Techniques Polytechnic College Al-Hawija Northern Technical University Kirkuk 36007 Iraq ntu.edu.iq; ^2^ Department of Prosthodontic Technology Institute of Health and Medical Technology Middle Technical University Baghdad Iraq mtu.edu.iq; ^3^ Department of Dental Technology Komar University of Science and Technology Sulaymaniyah Kurdistan Region Iraq komar.edu.iq

**Keywords:** 3D printing, CAD/CAM, digitally fabricated prostheses, facial disfigurement, maxillofacial prosthetics, prosthodontics, psychosocial rehabilitation, quality-of-life

## Abstract

This research sought to assess the psychological and social impacts of contemporary technology‐driven maxillofacial prosthetics in comparison to traditional counterparts. A comparative investigation was performed on 60 patients rehabilitated with digital *n* = 30 and traditional *n* = 30 prostheses at Ghazi Al‐Hariri Hospital for Surgical Specialties, Iraq. Data were acquired using the Hospital Anxiety and Depression Scale (HADS), a modified University of Washington Quality‐of‐Life (UW‐QOL)‐derived questionnaire, and a patient satisfaction questionnaire. SPSS v26 was used to perform descriptive statistics, independent *t*‐tests, Pearson correlations, and regression analysis. The significance level was set at *p* < 0.05. Patients rehabilitated with digitally fabricated prostheses reported significantly lower anxiety scores 5.70 vs. 6.97, *p* = 0.002 and depression scores 9.27 vs. 12.17, *p* < 0.001 than those rehabilitated with conventional prostheses. Quality‐of‐life scores were significantly higher in the appearance, social interaction, and emotional well‐being domains, overall mean = 4.3 vs. 3.5, *p* < 0.001. Users of digitally fabricated prostheses also reported significantly higher patient satisfaction, 4.6 vs. 3.8, *p* < 0.001. Patient satisfaction was positively correlated with quality oflife, *r* = 0.72, *p* < 0.001, and negatively correlated with anxiety, *r* = –0.68, *p* < 0.001. Hierarchical multiple regression analysis identified prosthesis type and overall patient satisfaction as significant predictors of quality of life, with the final model explaining 61.0% of the variance in modified UW‐QOL scores *R* ^2^ = 0.61, *p* < 0.001. Contemporary digital maxillofacial prosthetics were associated with better patient‐reported psychological health, esthetic self‐assurance, and social reintegration compared to conventional methods. It is important to combine digital processes, psychological therapy, and cooperation across different fields.

## 1. Introduction

Maxillofacial deformities, whether congenital, traumatic, or post‐oncologic, profoundly affect patients’ psychological health and social functioning. Beyond physical limitations in speech, mastication, and breathing, facial disfigurement undermines identity and self‐esteem, often leading to anxiety, depression, and social withdrawal [1]. The face is the main way people communicate with each other and show their feelings; thus, changes in the way the face looks may have a big impact on how patients see themselves and how they interact with other people [2].

Traditional craniofacial prostheses restore basic function but do not frequently meet the expectations of patients for vibrant and emotional esthetics. Some limitations in the range of colors and surface texture or in anatomical accuracy limit patient satisfaction and may impair psychosocial recovery [3], with many patients feeling stigmatized and psychologically distressed while receiving treatment [4].

With the introduction of new digital technologies like CAD/CAM systems, 3D printing, and facial scanning, highly individualized and anatomically realistic prostheses are now possible to create [5]. With these digital tools, it is possible to produce very individualized, anatomically realistic, and appearance‐natural prostheses. They also reduced the time they took to make them, fitted them better, and more closely matched the color [6].

These innovations offer a greater fit, color matching, and increased production efficiency. Importantly, they not only improve the appearance of the face but also offer emotional stability, social confidence, and a better quality of life [7]. Even with these advancements in technology, patients who suffer from a maxillofacial defect still experience a set of challenges that are not experienced by the majority of people [8].

Facial disfigurement can affect one’s sense of self and body image, resulting in stigma, social isolation, and loss of self confidence. The face is a focal point of interpersonal communication and emotional expression, unlike other diseases, so that changes in the appearance of the face can have a serious impact on interpersonal communication and emotional stability, which leads to a decrease in the ability to be integrated into society. These difficulties emphasize the critical need to assess functional and psychological aspects of prosthetic rehabilitation as well as social well‐being. Furthermore, individual differences in depression history, coping, and social support impact psychological adjustment [9].

Disparities in patient care could be further entrenched in resource‐poor areas due to unequal access to digital technology [10]. However, handmade production demands large amounts of material and energy resources [11]. Digital workflows like CAD/CAM and 3D printing cut waste and maximize the use of medical silicone [12].

This research aims to assess the psychological and social aspects of patients rehabilitated with conventional and digitally fabricated prostheses, focusing on their quality of life, emotional well‐being, and social reintegration. The study focuses on associations, not on causation, and offers evidence for helping to develop patient‐centered rehabilitation strategies.

The null hypothesis (H_0_) used was that there would be no significant difference between the psychological well‐being in people with conventional maxillofacial prostheses and those with digitally fabricated prostheses, social reintegration, and patient satisfaction. The alternative hypothesis (H_1_) was that patients receiving digitally fabricated prostheses would have significantly lower anxiety and depression scores, higher scores for quality‐of‐life, and greater satisfaction than those receiving conventional prostheses.

## 2. Materials and Methods

### 2.1. Study Design

This study employed a comparative design to evaluate the psychological and social impacts of maxillofacial prosthesis rehabilitation. Participants were divided into two groups: digital versus conventional prostheses, allowing a direct comparison of outcomes between fabrication methods. The research used a structured self‐administered questionnaire as the principal instrument for data collection [13].

### 2.2. Sample Size Calculation

The sample size was estimated a priori using 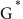
Power software (version 3.1.9.7), applying an independent samples *t*‐test (two‐tailed). For the analysis, an alpha level of 0.05, a power of 0.80, a 1−*β* ratio allocation of 1:1, and a large effect size Cohen’s *d* = 0.80 were used. was considered due to past comparative literature comparing the digital workflow and patient satisfaction in terms of craniofacial rehabilitation that had always shown a massive clinical improvement in esthetic and functional aspects while moving from manual to digital workflows [14, 15]. As a result of such estimation, the minimum sample size needed was estimated to be 52 participants (26 patients per group). To increase the power of statistics and to consider the possible non‐response rate, a total of 60 participants were included in the study (30 patients per group).

### 2.3. Study Setting and Participants

The research was conducted at Ghazi Al‐Hariri Hospital for Surgical Specialties, Iraq, from January to March 2026. Sixty patients who had undergone maxillofacial prosthesis rehabilitation were selected through purposive sampling and assigned to two comparative groups: digitally fabricated prostheses (*n* = 30) and conventional prostheses (*n* = 30). The assignment of patients to these groups was an observational one, dependent upon the particular mode of rehabilitation that the patients had undergone in regular clinical practice. To address potential clinical confounders, certain treatment variables were made constant; both groups underwent similar methods of retention, either a medical adhesive or a spectacle frame, according to the location of the defect, and all prostheses were fabricated using the same grade of medical silicone (MDX4‐4210). Facial scanning was performed with a handheld 3D optical scanner (3Shape intra oral scanner—Denmark), digital design was conducted using design software (Exocad—Germany) to reconstruct anatomical contours and symmetry, The molds were fabricated using a stereolithography (SLA) 3D printer (Formlabs Form 3, USA) with biocompatible resin, and a medical‐grade silicone (MDX4‐4210, Dow Corning, USA) was used for the final prosthesis, pigmented with intrinsic and extrinsic coloring techniques to match patient skin tones. Retention was achieved using medical adhesives (Pros‐Aide, USA) or spectacle frame support, depending on the defect site. Clinical chairside time averaged 2–3 sessions, while laboratory fabrication required ~12–15 h. All prostheses were fabricated by a prosthodontist with over 5 years of experience in digital workflows.

Conventional group patients were rehabilitated using traditional manual techniques. Impressions were taken with an irreversible hydrocolloid, followed by gypsum casts. Wax patterns were sculpted manually to approximate the facial anatomy. Final prostheses were fabricated with medical‐grade silicone (MDX4‐4210, Dow Corning, USA) and colored using extrinsic painting methods. Retention was achieved with adhesives or spectacle frame support. Clinical chairside time averaged 4–5 sessions, while laboratory fabrication required ~20–25 h. Prostheses were fabricated by experienced maxillofacial prosthodontists with more than 10 years of clinical practice.

Inclusion criteria were patients aged 18 years and above who had undergone maxillofacial prosthesis rehabilitation and consented to participate. Eligible patients were required to have clearly documented maxillofacial defects, including congenital, traumatic, or post‐oncologic etiologies, with specification of defect type (e.g., orbital, nasal, and auricular) and location. Patients with a history of reconstructive surgery were included, provided that the prosthesis was the primary mode of rehabilitation.

Exclusion criteria included patients with incomplete clinical records, those unwilling to participate, and individuals with confounding medical histories that could significantly influence psychosocial outcomes, such as prior radiotherapy or chemotherapy to the maxillofacial region, uncontrolled psychiatric illness, or systemic conditions impairing communication and social interaction.

In addition, patients with any history of psychological support prior to the study, including those with prior use of maxillofacial prosthesis, were excluded so that the psychosocial results obtained from the study could only be attributed to their current prosthetic rehabilitation process.

### 2.4. Research Tool

Using a standardized, self‐administered questionnaire with four main parts, we gathered data. Demographic and clinical information included age, gender, the reason and location of the facial deformity, the type of prosthesis obtained, the time elapsed since the fitting of the prosthesis, and employment status as an indication of socioeconomic status. All demographics and clinical factors obtained were recorded successfully and incorporated into the final analysis for group comparison.

The emotional well‐being was assessed with the Hospital Anxiety and Depression Scale (HADS), which comprised 14 questions, 7 for anxiety and 7 for depression, each scored on a 4‐point Likert scale (0–3), with a subscale range of 0–21. As per the original recommendations of the HADS, a score range of 0–7 is considered within normal limits, 8–10 represents a borderline abnormal level (possible case), and 11–21 represents a clinically significant level of anxiety or depression (probable case). A score of above 8 was a sign that the individual had clinically significant levels of anxiety or depression symptoms [16].

The Arabic version of the HADS, which was pre‐validated by El‐Rufaie and Absood [17], was used for linguistic and clinical validation. The pilot study showed good internal consistency of the anxiety and depression subscales of the Arabic‐translated HADS with Cronbach’s alpha (*α*) of 0.83 and 0.81, respectively.

The quality‐of‐life was assessed based on the University of Washington Quality‐of‐Life Questionnaire (UW‐QOL Version 4). Relevant domains to maxillofacial prosthetic rehabilitation were selected and adapted into a simplified 5‐point Likert scale for better patient understanding. The revised questionnaire evaluated the aspects of appearance, emotional health, and social interaction. Scores were derived as the mean across the items of the questionnaires and not based on the original UW‐QOL scoring system (0–100). Thus, the results are to be interpreted as adjusted QOL scores and should not be directly compared with studies that have been conducted with the original UW‐QOL scoring system. This adaptation was pilot‐tested for clarity and internal consistency [17], and during the pilot testing, a Cronbach’s alpha (*α*) of 0.85 was obtained.

Along with standardized scales (HADS and UW‐QOL), we created a patient satisfaction questionnaire based on relevant items from the facial disability index [18] and translated them to Arabic with the addition of prosthesis‐specific domains (color matching, comfort, fit, and functionality). In order to determine clarity and reliability, the questionnaire was piloted on 10 patients, with a Cronbach’s *α* value of 0.87, which is a high value of internal consistency.

In a pilot study, the Arabic tools proved to be very consistent internally, as measured individually for subscales. Arabic HADS Cronbach’s alpha was α = 0.83 for the anxiety subscale and α = 0.81 for the depression subscale. adapted Arabic UW‐QOL All subscales proved to be reliable, with values of appearance (α = 0.84), social interaction (α = 0.82), and emotional well‐being (α = 0.85). The patient satisfaction questionnaire Cronbach’s alpha for the whole scale was α = 0.87, whereas the subscales showed values of color matching (α = 0.85) and comfort and fit (α = 0.8). This measure has two components: bodily and social/well‐being functionality. The various sections are on a 5‐point scale [19].

The questionnaire was translated into Arabic and then back into English to make sure that it was correct. Translation involved a formal procedure that involved first translating the scales from English to Arabic by two independent bilingual prosthodontists; differences in translation were sorted out, and then an independent professional translator did back‐translation to English. Three prosthodontists and two clinical psychologists made up an expert panel that checked the face and content validity. This panel validated semantic, conceptual, and cultural equivalence for the adapted measures and provided an S‐CVI of 0.92. In addition, in order to examine reproducibility and temporal consistency, test–retest reliability was conducted in 10 pilot subjects at a 2 week interval, showing high consistency with ICCs from 0.82 to 0.89.

### 2.5. Data Collection

Before they filled out the questionnaire, participants were told about the study’s goals and how their privacy would be protected. Responses were gathered in person at follow‐up sessions, around 3 months after the delivery of the prosthesis, to confirm that participants had sufficient time to adjust. It took around 15–20 min to fill out each form.

### 2.6. Data Analysis

IBM SPSS Statistics version 26 (IBM Corp., Armonk, NY, USA) was used for statistical analysis. Continuous variables were summarized as mean ± standard deviation (SD), whereas categorical variables were presented as frequencies and percentages. Normality was assessed using the Shapiro–Wilk test, and homogeneity of variances was evaluated using the Levene’s test. In order to compensate for non‐random allocation and group equivalency, baseline demographic and clinical features (age, gender, cause of defect, and location of defect) have been analyzed using independent sample *t*‐tests and chi‐square tests (Table 1). Independent‐samples *t*‐tests were used to compare continuous variables between groups. Before conducting any parametric tests, the assumption of normality for all continuous variables was checked through the use of the Shapiro–Wilk test; all scales had *p* > 0.05, thus confirming their normal distribution. The assumption of homogeneity of variances was checked via Levene’s test, and in cases where the assumption of equal variances could not be assumed, Welch’s *t*‐test was used. Due to an increased risk of Type I error from multiple comparisons between UW‐QOL and patient satisfaction measures on different domains (4 domains for each variable), a Bonferroni correction *α* = 0.0125 (0.05/4) for the secondary comparisons of the subscales) was conducted in order to adjust the significance level. For the primary outcomes, significance was set at *p* < 0.05. For all comparisons, the *p*‐value, 95% confidence interval of the difference (95% CI), and the size of the differences (small: *d* = 0.2, medium: *d* = 0.5, and large: *d* ≥ 0.8) were determined. Categorical variables, such as HADS clinical categories, were compared via Pearson’s test.

**Table 1:** Baseline comparability of demographic and clinical characteristics between groups.

Variable	Conventional (*n* = 30) *n* (%) or mean ± SD	Digital *n* = 30 *n* (%) or mean ± SD	Statistical test	Exact *p*‐value
Age (years)	42.9 ± 11.7	42.7 ± 11.5	Independent *t*‐test (*t* = 0.07)	0.94
Gender	—	—	Chi‐square (*χ* ^2^ = 0.00)	1.00
Male	16 (53.3%)	16 (53.3%)	—	—
Female	14 (46.7%)	14 (46.7%)	—	—
Etiology of defect	—	—	Multi‐category *χ* ^2^ (*χ* ^2^ = 0.09)	0.96
Trauma	11 (36.7%)	11 (36.7%)	—	—
Post‐surgical (tumor)	13 (43.3%)	12 (40.0%)	—	—
Congenital	6 (20.0%)	7 (23.3%)	—	—
Site of defect	—	—	Multi‐category *χ* ^2^ (*χ* ^2^ = 0.12)	0.99
Orbital	10 (33.3%)	10 (33.3%)	—	—
Nasal	9 (30.0%)	8 (26.7%)	—	—
Auricular	7 (23.3%)	8 (26.7%)	—	—
Combined	4 (13.4%)	4 (13.4%)	—	—
Defect extent	—	—	Chi‐square (*χ* ^2^ = 0.00)	1.00
Single structure	26 (86.7%)	26 (86.7%)	—	—
Combined structures	4 (13.3%)	4 (13.3%)	—	—
Duration since delivery	—	—	Multi‐category *χ* ^2^ (*χ* ^2^ = 0.36)	0.84
<3 months	8 (26.7%)	6 (20.0%)	—	—
3–6 months	15 (50.0%)	17 (56.7%)	—	—
>6 months	7 (23.3%)	7 (23.3%)	—	—
Reconstructive surgery	—	—	Chi‐square (*χ* ^2^ = 0.07)	0.79
Yes	12 (40.0%)	11 (36.7%)	—	—
No	18 (60.0%)	19 (63.3%)	—	—
Employment status	—	—	Multi‐category *χ* ^2^ (*χ* ^2^ = 0.33)	0.95
Employed	13 (43.3%)	15 (50.0%)	—	—
Unemployed	10 (33.3%)	8 (26.7%)	—	—
Retired	5 (16.7%)	5 (16.7%)	—	—
Student	2 (6.7%)	2 (6.7%)	—	—

For the purpose of determining the independent predictors of UW‐QOL and considering possible additional independent predictors, hierarchical multiple linear regression analyses were conducted. In model one, the main predictor of interest, Prothesis Type (0 for conventional and 1 for digital), and baseline demographics (age and gender) were included. The second model included overall patient satisfaction in order to consider it as an additional independent predictor.

In preparation for the regression analyses, the following main assumptions were tested:

1. Tolerance and variance inflation factor (VIF) values were used to check multicollinearity; VIF < 1.5 means absence of collinearity.
2. Breusch–Pagan test for homoscedasticity was conducted, *p* = 0.28.
3. Normality of residuals was checked by the Shapiro–Wilk test (*p* = 0.42) and Normal P–P plots were constructed.

The statistical significance level was set at α = 0.05.

### 2.7. Ethical Considerations

The ethical research committee of Northern Technical University approved the research (Approval No. Ref: 6). Participation was optional, and informed written permission was obtained. Throughout the research, confidentiality and anonymity were preserved, and individuals in difficulty were sent for psychological assistance. As this was an observational comparative study and did not involve prospective assignment of participants to healthcare interventions, clinical trial registration was not applicable.

## 3. Results

### 3.1. Demographic Characteristics of Participants

Table 2 presents the demographic and clinical characteristics of the study participants, including age, gender, etiology, and location of the maxillofacial defects, as well as the type of prosthesis obtained, duration since prosthesis delivery, and employment status.

**Table 2:** Demographic and clinical characteristics of participants (*N* = 60).

Variable	Category	*n* (%) or mean ± SD
Gender	Male	32 (53.3%)
	Female	28 (46.7%)
Age (years)	—	42.8 ± 11.6
Etiology of defect	Trauma	22 (36.7%)
	Post‐surgical (tumor)	25 (41.7%)
	Congenital	13 (21.6%)
Site of defect	Orbital	20 (33.3%)
	Nasal	17 (28.3%)
	Auricular	15 (25.0%)
	Combined	8 (13.3%)
Defect extent	Single structure	52 (86.7%)
	Combined structures (mixed)	8 (13.3%)
Duration since delivery	<3 months	14 (23.3%)
	3–6 months	32 (53.3%)
	>6 months	14 (23.3%)
Reconstructive surgery	Yes (history of reconstruction)	23 (38.3%)
	No	37 (61.7%)
Employment status	Employed	28 (46.7%)
	Unemployed	18 (30.0%)
	Retired	10 (16.7%)
	Student	4 (6.7%)

*Note*: Data are presented as *n* (%) for categorical variables and mean ± SD for continuous variables; the age range was 21–67 years.

Sixty participants filled out the research questionnaire, 32 of whom were men 53.3% and 28 of whom were women 46.7%. The average age of the participants who took part was 42.8 years, with ages ranging from 21 to 67. In terms of the cause of the problems, 36.7% of patients had posttraumatic defects, 41.7% had surgical resections after tumor removal, and 21.6% had congenital malformations. The defect locations were as follows: orbital 33.3%, nasal 28.3%, auricular 25.0%, and mixed facial abnormalities 13.4%. As far as the period of prosthesis use clinically is concerned, 53.3% of participants had worn it between 3 and 6 months, whereas 23.3% had worn it for less than 3 months and 23.3% had worn it for more than 6 months. As far as employment/socioeconomic class is concerned, 46.7% were employed, 30.0% were unemployed, 16.7% were retired, and 6.7% were students.

#### 3.1.1. Baseline Comparability Between Groups

Independent sample *t*‐tests and chi‐square tests were conducted to evaluate baseline equivalence between the digital and conventional groups regarding age, gender distribution, etiology, and defect site, as shown in Table 1.

Baseline demographic and clinical characteristics were comparable between the conventional and digitally fabricated prosthesis groups, revealing no statistically significant differences between groups in age, gender distribution, defect etiology, or defect site *p* > 0.05, confirming adequate baseline equivalence prior to outcome comparisons.

### 3.2. Psychological Well‐Being HADS Scores

The mean level of anxiety and depression in patients who were rehabilitated with the help of digitally fabricated prostheses was significantly lower in comparison with the traditional ones. The digitally fabricated prosthesis group demonstrated significantly lower anxiety scores than the conventional group, 5.70 ± 2.02 vs. 6.97 ± 0.61, *p* = 0.002. Likewise, depression scores were significantly lower in the digital group, 9.27 ± 1.23 vs. 12.17 ± 1.18, *p* < 0.001, as shown in Table 3.

**Table 3:** Psychological well‐being score comparisons.

Parameter (HADS)	Conventional (*n* = 30) mean ± SD	Digital (*n* = 30) mean ± SD	*t*‐Value	Exact *p*‐value	95% CI of the difference	Levene’s test (*p*‐value)
Anxiety score	6.97 ± 0.61	5.70 ± 2.02	3.286	0.002	0.484−2.050	<0.001
Depression score	12.17 ± 1.18	9.27 ± 1.23	9.331	<0.001	2.278–3.522	0.536

Digital prosthetic rehabilitation was associated with markedly reduced anxiety and depression levels, reflecting better emotional adjustment and psychological recovery.

### 3.3. Social Reintegration and Quality‐of‐Life

Patients rehabilitated with digitally fabricated prostheses reported higher satisfaction across adapted UW‐QOL domains. Using the modified 1–5 Likert scoring, digital users had a mean overall score of 4.3 compared to 3.5 in the conventional group (*p* < 0.01). The most improved domains were appearance satisfaction, emotional well‐being, and social interaction (adapted domain), as shown in Table 4.

**Table 4:** Comparative analysis of the UW‐QOL domains.

UW‐QOL domain	Conventional (*n* = 30) mean ± SD	Digital (*n* = 30) mean ± SD	*t*‐Value (df = 58)	Exact *p*‐value (unadjusted)	Cohen’s *d*	95% CI of the difference	Bonferroni significance (threshold: 0.0125)
Appearance	3.6 ± 0.7	4.5 ± 0.5	5.73	<0.001	1.48	0.59−1.21	Significant
Social interaction	3.4 ± 0.6	4.2 ± 0.4	6.08	<0.001	1.57	0.54−1.06	Significant
Emotional well‐being	3.3 ± 0.7	4.3 ± 0.5	6.37	<0.001	1.64	0.69−1.31	Significant
Overall QOL score	3.5 ± 0.6	4.3 ± 0.4	6.08	<0.001	1.57	0.54−1.06	Significant

### 3.4. Esthetic and Functional Satisfaction

Those who used digitally fabricated prostheses reported higher satisfaction with appearance, comfort, and functionality. Using our validated satisfaction questionnaire, digital users had an average score of 4.6 compared to 3.8 in the conventional group (*p* < 0.001). The most improved areas were comfort, color matching, and tolerance of wear duration, as shown in Table 5.

**Table 5:** Esthetic and functional satisfaction scores.

Satisfaction domain	Conventional (*n* = 30) Mean ± SD	Digital (*n* = 30) Mean ± SD	*t*‐Value (df = 58)	Exact *p*‐value (unadjusted)	Cohen’s *d*	95% CI of the difference	Bonferroni significance (Threshold: 0.0125)
Color matching	3.7 ± 0.6	4.7 ± 0.3	8.16	<0.001	2.11	0.75−1.25	Significant
Comfort and fit	3.8 ± 0.7	4.6 ± 0.4	5.44	<0.001	1.41	0.51–1.09	Significant
Functionality	3.6 ± 0.8	4.5 ± 0.5	5.21	<0.001	1.35	0.55−1.25	Significant
Overall satisfaction	3.8 ± 0.5	4.6 ± 0.3	7.51	<0.001	1.94	0.59−1.01	Significant

### 3.5. Correlation Analysis

Pearson correlation analysis showed that there was a strong positive correlation between the patient satisfaction questionnaire and quality‐of‐life (UW‐QOL) *r* = 0.72, *p* < 0.001. It was found that the levels of anxiety had a strong negative correlation with both satisfaction *r* = −0.68, *p* < 0.001) and quality‐of‐life ratings *r* = −0.64, *p* < 0.001, which implies that a higher level of esthetics and functionality is directly connected with a lower level of psychological discomfort.

### 3.6. Regression Analysis

Hierarchical multiple regression analysis was conducted to predict the outcome variable (quality‐of‐life, UW‐QOL) using the independent variable, prosthesis type, as well as demographic variables and patient satisfaction. Table 6 provides the entire set of coefficients.

**Table 6:** Linear regression predicting overall quality of life (UW‐QOL).

Predictor variables	Unstandardized *B*	Slandered error	Standardized *B*	*t*‐statistic	*p*‐Value	95% confidence interval (CI)	Collinearity (VIF)
Model 1							
Constant	3.65	0.32	—	11.41	<0.001	3.01, 4.29	—
Prosthesis type 0 = Conv, 1 = Dig)	0.80	0.12	0.68	6.67	<0.001	0.56, 1.04	1.00
Age (years)	−0.002	0.005	−0.04	−0.40	0.69	−0.012, 0.008	1.01
Gender (0 = Fem, 1 = Male)	0.02	0.11	0.02	0.18	0.86	−0.20, 0.24	1.01
Model 2							
Constant	1.85	0.45	—	4.11	<0.001	0.95, 2.75	—
Prosthesis type (0 = Conv, 1 = Dig)	0.42	0.14	0.36	3.00	0.004	0.14, 0.70	1.46
Overall satisfaction	0.48	0.11	0.47	4.36	<0.001	0.26, 0.70	1.45
Age (years)	−0.001	0.004	−0.02	−0.25	0.80	−0.009, 0.007	1.01
Gender (0 = female, 1 = male)	0.01	0.10	0.01	0.10	0.92	−0.19, 0.21	1.01

*Note:* Model 1: *R* ^2^ = 0.46, Adjusted *R* ^2^ = 0.43, *F*(3,56) = 15.93, *p* < 0.001; Model 2: *R* ^2^ = 0.61, Adjusted *R* ^2^ = 0.58, *F*(4,55) = 21.50, *p* < 0.001; *Model change:* Δ*R* ^2^ = 0.15, F‐change = 21.13, *p* < 0.001.

Model 1 was done, where the prosthesis type was the main predictor, together with age and gender. The model was statistically significant, *F* (3,56) = 15.93,*p* < 0.001, and explained 46.0% of the variability in quality of life, *R* ^2^ = 0.46, adjusted *R* ^2^ = 0.43. With other demographics held constant, patients who underwent rehabilitation using digitally fabricated prostheses had a significantly higher quality of life than those with conventional prostheses *B* = 0.80, SE = 0.12, *β* = 0.68,*p* < 0.01.

In Model 2, overall patient satisfaction was entered into the regression equation. This led to a significant increase in the proportion of variance accounted for, Δ*R* ^2^ = 0.15, F change = 21.13, *p* < 0.001, with the model as a whole accounting for 61.0% of variance in quality‐of‐life, *R* ^2^ = 0.61, Adjusted *R* ^2^ = 0.58, *F* (4,55) = 21.50, *p* < 0.001. Overall patient satisfaction was a highly significant independent predictor of quality‐of‐life, *B* = 0.48, SE = 0.11, *β* = 0.47, *p* < 0.00,95%, CI: 0.26, 0.70, even after taking into consideration, after adjustment for overall patient satisfaction, the main exposure variable, prosthesis type, still remained a highly significant variable (Table 6).

## 4. Discussion

### 4.1. Key Findings

This comparative study found that there was a significant correlation between digitally fabricated prostheses manufacturing and better psychological and social outcomes. Nonetheless, there can be no causality as there is an observational design. The digital group was less anxious and less sad (HADS) and more socially and emotionally satisfied with life (UW‐QOL). The patient satisfaction questionnaire results indicated that digitally fabricated prostheses received improved evaluations in color, comfort, and function. The combination of these results suggests that technology innovation is associated with better psychosocial restoration and an improved self‐image among patients after facial damage.

Statistically significant changes in the state of anxiety, depression, and QoL in patients from the group receiving prostheses produced via digital methods could be scientifically substantiated by the advanced clinical and physical parameters of the new technology compared to traditional ones.

### 4.2. Mechanistic Interpretation

From the biomechanics point of view, hand‐carving of wax patterns is affected by the human factor and dimensional reduction of dental stone molds and silicone due to their own properties [20]. The advantages of digital workflows in patient‐specific design, customization, and fabrication could be linked to the improved comfort and esthetic satisfaction experienced with the digitally fabricated prostheses [12, 20, 21]. Previous clinical literature also indicates that prosthesis‐related factors, including anatomical fit, color, shape, and comfort of wearing prosthesis, are closely linked with patient satisfaction [14], which in the current study was associated with the enhanced psychosocial outcomes. This interpretation is further supported by the increasing clinical interest in the comparison of digital and conventional processes for the facial prosthesis workflow [15]. Physically, this allows for producing prosthesis with margins being very thin, feather‐like, and perfectly adapted to biological margins of the defect. This high margin adaptation reduces the gap between the skin and the silicone and prevents the appearance of air pockets, which improves the natural mechanical retention of the prosthesis [22].

Moreover, the use of 3D‐molded prosthetic implants assures a consistently high uniformity of thickness of the medical‐grade silicone [23]. The higher physical comfort reported by the digital group 4.6 ± 0.3 vs. 3.8 ± 0.5, *p* < 0.001, may be related to improved prosthesis fit and patient‐centered characteristics associated with digital fabrication. Previous studies have reported that prosthesis fit and wearing comfort are important determinants of patient satisfaction with maxillofacial prostheses [14], while digital workflows have demonstrated acceptable fit and improved patient comfort and satisfaction. When the prosthesis is both physically comfortable and mechanically stable, the patients feel safe from the somatic point of view; they are not afraid anymore to lose their prosthesis in public places due to accidental falling off during movement [9].

The physical security thus immediately triggers a positive psychological chain reaction. Facial deformity severely affects the body image and self‐concept, leading to cognitive avoidance and depression [24]. Digitally fabricated prostheses have managed to overcome the problem with conventional prostheses that fail to achieve realistic visual results and avoid entering the uncanny valley phenomenon [25]. In turn, the use of a digitally fabricated prosthesis allows overcoming the body image distortion issue by making it nearly imperceptible during social interaction. The decrease in HADS scores anxiety 5.70 ± 2.02 vs. 6.97 ± 0.61, depression 9.27 ± 1.23 vs. 12.17 ± 1.18 is caused by the disruption of the psychological distress cycle. With a feeling of physical security, patients no longer need to constantly monitor themselves; thus, the patient receives an opportunity for immediate social reintegration. Instead of withdrawing from social interactions due to the threat of stigmatization [26], individuals who underwent rehabilitation through digital technology regain confidence in their verbal and nonverbal interactions. The substantial increase in UW‐QOL score in the social interaction domain 4.2 ± 0.4 vs. 3.4 ± 0.6, *p* < 0.01 proves that better marginal fit of the prosthetic material and its high degree of realism make patients more confident in participating in family celebrations, speeches, etc., without embarrassment [2, 7].

### 4.3. Comparison with Previous Literature

The current study’s patient satisfaction results indicate that digitally fabricated prostheses received improved evaluations in color, comfort, and function, aligning with the findings of Bibb et al. [27], which established that additive manufacturing guarantees constant accuracy and enhanced color matching. Cristache et al. [20] corroborated that digital processes improve fit accuracy and patient comfort, essential components reflected in our findings.

Fayad et al. [25] underscored that the amalgamation of digital coloring and 3D modeling markedly enhanced esthetic realism and diminished production time, results corroborated by our participants’ increased contentment and alleviated emotional stress. In the same way, Prakash et al. [28] observed that contentment with one’s appearance is a substantial factor in psychological well‐being. This supports the positive link we established in our regression model between patient satisfaction and UW‐QOL scores.

The results show that there is a direct effect of technological growth on psychological recovery. CAD/CAM and 3D printing’s accuracy and customization help reduce the visibility of prostheses, enhance symmetry, and restore the self‐image, which contributes to anxiety and depression. These results again highlight the importance of multi‐specialty rehabilitation, which combines the developments in prosthetics with psychological support programs [29–31].

Digital processes could reduce the time required to produce products in clinical practice and the number of chairside patient visits. Improve appearance and psychological well‐being, strengthen communication between the patient and the practitioner, and individualize the face’s characteristics. However, access to these is still not an issue in low‐resource areas where digital fabrication is costly, and ensuring that everyone has equal access remains a challenge. Evidence shows that digital maxillofacial prosthesis rehabilitation is considered a more effective solution to improve patients’ psychological and social status when compared with traditional rehabilitation methods, which could help fill this gap, if increased training in digital prosthodontics is offered and financial aid is provided for the purchase of technology. The results highlight the importance of combining the accuracy of technology with a holistic approach to patient‐centered care, ensuring physical recovery, and emotional reintegration.

## 5. Limitations

A few restrictions need to be recognized regarding the interpretation of the current results; the design is observational and no randomization has taken place, and causal inference about the relationship between fabrication workflow and psychosocial outcomes is not possible. The groups were similar in the demographic and clinical variables measured, but residual and unmeasured confounding factors cannot be ruled out. Psychological and quality‐of‐life evaluations were not done prior to prosthetic rehabilitation. Therefore, no within‐patient differences from baseline were possible, and the changes in psychological adjustment that occurred prior to the study may have affected the results. The relatively brief follow‐up time precludes drawing conclusions about the sustainability of the psychological, social, and satisfaction results. The performance of the prosthesis and its adaptation to the psychosocial environment can also vary over time, depending on the properties of the material and the adaptation of the patients to the prostheses.

The participants knew what kind of technique was used to make their prostheses. Patients who received a digitally fabricated prosthesis might have higher expectations for new technology, which may have affected the self‐reported prosthesis satisfaction and quality‐of‐life outcomes, expectancy, or placebo effects. The study was performed in one treatment facility and included a relatively small purposive sample. Thus, the results might not apply to other populations, health care systems, defects, or centers with varying levels of digital expertise and resources. Furthermore, the Arabic instruments used in the adaptation were found to be internally consistent and have a good test–retest reliability in the pilot testing but not fully psychometric validated. The present results must be considered as hypothesis‐generating observation evidence and not as proof of which of the two fabrication methods is superior. Further studies to verify these relationships and evaluate the efficacy of digitally fabricated maxillofacial prostheses for long‐term improvements in psychosocial outcomes would benefit from multicenter prospective studies and adequately powered randomized controlled trials with baseline assessment and longer follow‐up periods.

### 5.1. Clinical Implications

Clinical importance of these results is enormous in contemporary maxillofacial rehabilitation. Not only does the shift from laborious laboratory techniques to digital procedures result in increased efficiency at the clinical chair average of 2–3 visits in comparison with 4–5 visits in the conventional group but also ensures predictable and reproducible psychological rehabilitation. In their practice, clinicians should consider digital maxillofacial prosthetics as much more than just a mechanical replacement technique; rather, it should be perceived as an instrument of primary psychosocial treatment.

## 6. Conclusion

This paper has provided observational evidence that patients rehabilitated with digitally fabricated maxillofacial prostheses reported better psychological function, esthetic satisfaction, and social reintegration compared to conventional techniques through the use of CAD/CAM design and 3D printing in providing better outcomes when compared to the conventional fabrication techniques, which improves both physical and psychological function, esthetic satisfaction, and social reintegration. Patients who had received digitally manufactured prostheses were much less anxious and hopeless, had a high level of self‐esteem, and felt better about their appearance, comfort, and the general quality of life.

Through a therapeutic perspective, holistic treatment of patients should not only be mechanical restoration but also psychological support, peer support, and multidisciplinary team care among prosthodontists, surgeons, and mental professionals. Further studies ought to entail longitudinal multicenter investigations that explore the long‐term permanence of psychological adaptability, explore the cost‐effectiveness of digital procedures, and research the use of artificial intelligence to offer greater customization and predictive modeling.

## Author Contributions

**Shahad Fadhil Bunyan:** conceptualization, methodology, supervision, formal analysis, writing – review and editing. **Shahad Mohammed Shakir:** conceptualization, investigation, data curation, formal analysis, writing – original draft. **Zryan Jaza Hama Salih:** investigation, data collection, resources and validation. **Wisam Jasim Mohammed:** statistical analysis, data collection, resources. **Quasy Abdulateef Hasan:** methodology, writing – original draft and data curation.

## Funding

No funding was received for this research.

## Disclosure

All authors have read and approved the final version of the manuscript. Shahad Fadhil Bunyan had full access to all of the data in this study and takes complete responsibility for the integrity of the data and the accuracy of the data analysis.

## Conflicts of Interest

The authors have no conflicts of interest.

## Data Availability

Data are available upon request from the authors.
